# Clinical Diagnostics After Failed Hearing Screening in People With Intellectual Disabilities Do Not Often Take Place

**DOI:** 10.1111/jir.70078

**Published:** 2026-01-16

**Authors:** Anna Wiegand, Philipp Mathmann, Susanne Wasmuth, Lukas Prein, Ross Parfitt, Martin Scharpenberg, Vincent Jankovic, Katharina Schwarze, Anja Neumann, Karolin Schäfer, Christian Speckemeier, Sven Baessler, Sarah Schlierenkamp, Sandra Diekmann, Nicole Stuhrmann, Ruth Lang‐Roth, Muhittin Demir, Werner Brannath, Awa Naghipour, Susanna Marie Zielonkowski, Anna Sophia Schwalen, Corinna Gietmann, Katrin Neumann

**Affiliations:** ^1^ Department of Phoniatrics and Paediatric Audiology University Hospital Münster Münster Germany; ^2^ Oberberg Fachklinik Potsdam Potsdam Germany; ^3^ Department of Otorhinolaryngology, Head and Neck Surgery Johannes Wesling Klinikum Minden, Ruhr‐University Bochum Minden Germany; ^4^ South African College of Music University of Cape Town Cape Town South Africa; ^5^ Competence Center for Clinical Trials Bremen University of Bremen Bremen Germany; ^6^ Institute for Health Care Management and Research University of Duisburg‐Essen Essen Germany; ^7^ Institute for Special Needs Education (d/Deaf and Hard of Hearing) University of Duisburg‐ Essen Germany; ^8^ Essener Forschungsinstitut für Medizinmanagement ‐ EsFoMed GmbH Essen Germany; ^9^ Practice for Otolaryngology, Phoniatrics & Paediatric Audiology Duesseldorf‐Meerbusch Germany; ^10^ Phoniatrics and Paediatric Audiology, Head and Neck Surgery, Helios HSK Wiesbaden Germany; ^11^ Department of Otorhinolaryngology, Head and Neck Surgery, Medical Faculty University Hospital Cologne Cologne Germany; ^12^ Department of Otorhinolaryngology, Division of Phoniatrics and Paediatric Audiology University Medicine Essen Essen Germany

**Keywords:** assessment, diagnostics, hearing loss, hearing screening, intellectual disabilities, programme

## Abstract

**Background:**

Individuals with intellectual disabilities are at higher risk of undiagnosed or inadequately treated hearing loss. This situation requires easily accessible hearing screening, diagnostics and intervention programmes in the living environment, i.e., in nurseries, schools, workplaces and homes. However, a full audiometric assessment is not always possible in nonclinical settings. The multicentre cohort study *HörGeist* investigated the effectiveness, feasibility and costs of an outreach programme of repeated hearing screening, diagnostics, intervention and monitoring of children, adolescents and adults with intellectual disabilities in their living environment in comparison with an invitation‐only programme comprising a control cohort in a clinical setting and with standard care. This paper reports on the HörGeist substudy of the outreach cohort, focusing on participants referred for ‘external’ diagnostics in clinical settings after failing on‐site screening, and evaluating both referral uptake and outcomes.

**Methods:**

Because none of the 141 individuals in the control cohort provided informed consent to attend the programme in a clinical setting, our results pertain solely to the outcomes and feasibility within the outreach cohort. All of the 1053 participants in the outreach cohort who failed the hearing screening tests underwent full on‐site audiometric assessment. Where on‐site screening and/or diagnostics were not feasible, referrals to external medical institutions were provided. Participants who were referred to external diagnostics were tracked via telephone interviews using a questionnaire and asked about their utilisation and the outcome of diagnostics. In cases where referrals were not pursued, reasons for non‐compliance were recorded.

**Results:**

A referral for external diagnostics was received by 262 of the 1053 participants of the outreach cohort. Of these, 19 dropped out of the study. Of the 248 referrals received by the remaining 243 participants, 93 (37.5%) were attended and 155 (62.5%) were not. The main reasons for non‐attendance were ‘no attempt to arrange an appointment’ (32.9%), ‘refusal by caregivers’ (23.2%) and ‘refusal by participants’ (18.1%). Approximately 4% did not receive an appointment for external diagnostics. Referral uptake declined with age, with uptake rates of 50.8% in young children, 41.3% in school‐aged participants and 24.7% in adults. Telephone tracking of a subsample of 48 participants who primarily did not attend for external assessment led to further clinical diagnostics in eight cases (16.7%).

**Conclusions:**

In order to achieve an improvement in the hearing situation of people with intellectual disabilities, a screening, diagnostic and intervention programme in their living environment seems both feasible and beneficial. However, reliable assessment of the hearing status of the participants of such a programme requires education of participants, caregivers and medical professionals regarding its necessity and fostering of close collaboration with healthcare providers in outpatient and clinical settings.

**Trial Registration:** German Clinical Trials Register (DRKS‐ID: DRKS00024804)

## 1 Introduction

Individuals with intellectual disabilities typically experience a range of health challenges. These include both syndrome‐specific comorbidities and lifestyle‐related conditions, such as cardiovascular, nutritional, dental and ophthalmic diseases (Hild et al. 2008). One such frequently overlooked and inadequately treated condition is hearing loss (Kumar Sinha et al. 2008; Haile et al. 2021; Willems et al. 2022). Hearing loss is typically classified as conductive, which may be temporary or fluctuating, or chronic up to permanent, caused by impaired sound transmission through the outer or middle ear; sensorineural, generally a permanent loss resulting from dysfunction of the cochlea or auditory nerve, affecting sound perception and processing; or mixed, which combines both conductive and sensorineural components and often includes a permanent element (Michels and Angeles 2019; Danyluk and Jacob 2023). The WHO's 2019 Burden of Disease Study identified hearing loss as the third leading cause of years of healthy life lost due to disability (Haile et al. 2021). Individuals with intellectual disabilities even have a significantly higher risk of experiencing persistent hearing loss than the general population (Evenhuis et al. 2001; Neumann, Dettmer, et al. 2006; Hey et al. 2014).

Many studies highlight the high prevalence of hearing loss in individuals with intellectual and developmental disabilities, with reported rates varying from 24% (Herer 2012) to 93% (Evenhuis et al. 2001), depending on factors such as age and whether individuals with Down syndrome are included. In particular, individuals with Down syndrome, who make up the largest proportion of people with intellectual disabilities (approx. 15% (Bower et al. 2000)), have a high prevalence of hearing loss (Esbensen 2010; Määttä et al. 2011; Malt et al. 2013; Kreicher et al. 2017). Typical anatomical features often lead to middle ear effusion, chronic ear infections, cerumen build‐up and related conductive hearing loss (Nightengale et al. 2017). Persistent hearing loss is reported in 21% to 50% of individuals with Down syndrome (Evenhuis et al. 2001; Heß et al. 2005). Age‐related hearing loss (presbycusis) also tends to develop in individuals with Down syndrome approximately 30 years earlier than in the general population (Meuwese‐Jongejeugd et al. 2006).

Hearing loss and other conditions such as poor dental health and sleep apnoea are often overlooked and either go untreated or are inadequately addressed in individuals with intellectual disability (Anders and Davis 2010; Lennox et al. 2011; Riha et al. 2024), which is why hearing loss is sometimes referred to as a hidden disability (Mackenzie and Smith 2009). Contributing factors include short consultation times (Lennox et al. 2005), which heighten distress in individuals with intellectual disabilities, and often lead to significant reductions in verbal communication abilities (Shady et al. 2022) and the reinforcing of existing communication barriers (García et al. 2020). When combined with limited access to healthcare information—such as the lack of easy‐to‐read materials—the process of shared decision‐making in healthcare is complicated further (Sullivan and Heng 2018). One key factor contributing to the high incidence of undiagnosed and untreated hearing loss among individuals with intellectual disabilities is the complexity of logistical and organisational challenges involved in accessing medical care (García et al. 2020). Typically, these individuals require the assistance of a caregiver, educator or family member to accompany them, and those with significant motor impairments may need specialised transportation to reach healthcare facilities (Savvas et al. 2025). Because individuals with intellectual disabilities rarely self‐report hearing difficulties, such problems may remain undetected in everyday social interactions (Evenhuis 1996), despite caregivers' and relatives' best efforts. Healthcare providers themselves face challenging conditions as well: healthcare professionals often express discomfort or report insufficient training in communicating with and providing care for individuals with intellectual and developmental disabilities, a fact that highlights potential challenges in ensuring these patients have access to high‐quality, appropriate care (Shady et al. 2022; Danyluk and Jacob 2023).

It is well‐known within paediatrics that diagnostic difficulties often lead to delayed diagnoses, which can have lasting effects on speech, language and cognitive development (Yoshinaga‐Itano et al. 1998; Neumann, Böttcher, and Euler 2006; Yoshinaga‐Itano et al. 2021). For example, children with intellectual disabilities have significantly less access to gold‐standard assessments than those without developmental disabilities and are therefore at high risk of receiving suboptimal hearing evaluations (Bonino et al. 2024). For individuals with intellectual disabilities of all ages, methods from paediatric audiology are often the only applicable ones. Furthermore, accurately determining the hearing threshold and the type of hearing loss (conductive, sensorineural and combined conductive‐sensorineural and central) often requires general anaesthesia in this population (Trudeau et al. 2021), which presents logistical challenges for both caregivers and clinicians.

Given the high prevalence of hearing loss among individuals with intellectual disabilities on one hand and inadequate diagnostics and treatment on the other, there is a clear need for low‐threshold, easily accessible hearing screening programmes (Bonino et al. 2024). Such programmes may have a positive effect on the health outcomes of individuals with intellectual disabilities and disease prevention and are more cost‐effective than standard care approaches (Lennox et al. 2011).

We developed an outpatient screening, diagnostic and intervention programme designed for individuals with intellectual disabilities that could be carried out in their living environments, such as workplaces, nurseries, schools and care homes. The aim was to conduct as much diagnostic work as possible on‐site. However, in the majority of cases, no diagnostics or only incomplete diagnostics could be performed. This was due to various factors, including the lack of availability of procedures such as imaging and non‐compliance by participants. This paper thus focuses on participants referred for external diagnostics after failing on‐site screening or assessment and evaluates the uptake of these referrals and the outcomes achieved.

## 2 Methods

The data presented here is part of the large cohort study *HörGeist*, which evaluated an outreach programme involving hearing screening, diagnostics, intervention and ongoing monitoring of individuals with intellectual disabilities in their living environment (outreach group). The same procedure was repeated 1 year later in order to examine the outcome of the programme. Outcomes were also compared to an invitation‐only programme conducted in a clinical setting (clinical group) and to standard care (Schwarze et al. 2023). In this report, we focus solely on the outreach group because only 12 of the 141 individuals invited for a clinical hearing assessment through their families attended the appointment, with none providing informed consent to participate in the study, and standard care was assessed only from a health‐economic perspective.

### 2.1 Participants

A total of 1053 individuals with intellectual disabilities of all ages who were insured by AOK Rheinland/Hamburg (the project's partner and Germany's largest health insurance provider) were recruited for the outreach group in the Rhineland region of Germany. Recruitment took place across 158 facilities that serve as living environments for people with intellectual disabilities, including nurseries, schools, workplaces and residential homes. Three age groups were defined: young children (0–5 years, *n* = 231), school‐aged children and adolescents (6–17 years, *n* = 405) and adults (18 years and older, *n* = 417). Enrollment began in September 2021 and concluded in June 2024. As part of a comprehensive medical history, the estimated severity of intellectual disability was also recorded. These severity estimates were based on caregiver assessments, which in some cases drew on medical records but were often informed by their professional judgement (Zielonkowski et al. 2025). The present analysis focuses on a subgroup (*n* = 262) drawn from the overall sample; further details on this cohort and the inclusion criteria are given in Section 2.2. Table 1 summarises the demographics of this subgroup.

**TABLE 1:** Demographics of the study cohort including age, sex, type of facility and caregiver‐reported severity of intellectual disability.

	Age group 1 (0–5)	Age group 2 (6–17)	Age group 3 (18–90 years)	Total
*N*	67	96	99	262
Age				
Mean	3,9	9,5	56,7	25,9
Standard deviation	1,1	3,1	14,8	25,9
Sex				
Male	42	68	50	160
Female	24	28	49	101
Diverse	1	0	0	1
Testing location				
Special educational nursery	39	4	0	43
Inclusive nursery	28	9	0	37
Children's residential group	0	0	0	0
Special needs school	0	78	5	83
Inclusive school	0	0	0	0
Youth residential group	0	5	2	7
Sheltered workshops	0	0	10	10
Company‐integrated workplaces	0	0	0	0
Adult residential group	0	0	74	74
Assisted living	0	0	8	8
Estimated degree of disability				
Mild	10	9	15	34
Moderate	28	26	45	99
Severe	22	58	29	109
Missing	7	3	10	20
Down syndrome				
Yes	8	17	19	44
No	59	79	80	218

*Note:* Please note that here, ‘diverse’ refers to biological sex, not gender identity.

Following approval from the Ethics Committee of the Medical Association of Westphalia‐Lippe and the University of Münster (No. 2020‐843‐f‐S), all participants—or their parents and/or legal guardians—received comprehensive study information, provided in Plain Language (clear and straightforward communication using simple words and structure) where necessary. Written consent was obtained, along with a confidentiality release for caregivers. The study was conducted in accordance with the Declaration of Helsinki and Good Clinical Practice (GCP) guidelines. The study is registered in the German Clinical Trials Register (DRKS‐ID: DRKS00024804).

### 2.2 Study Design

Outreach screening was conducted in nurseries, day care centres, schools, residential facilities and workplaces, depending upon the age group. Assessments were conducted at the first visit (time point T0) and approximately 12 months later (T1), with subsequent diagnostic and/or therapeutic procedures followed up as needed at both time points. If participants failed the screen, a comprehensive diagnostic assessment was promptly conducted, and where required, an intervention was implemented or an existing therapy, such as the use of hearing aids, was monitored.

The screening programme comprised a multistep hearing assessment, beginning with otoscopy, measurement of transient‐evoked otoacoustic emissions (TEOAE), tympanometry and pure‐tone audiometry (PTA) screening at frequencies 0.5, 1.0, 2.0, 4.0, and 8.0 kHz—either through conventional PTA or the interactive adaptive self‐test Multiple‐choice Auditory Graphic Interactive Check (MAGIC; PATH MEDICAL GmbH). If PTA was not feasible, distortion‐product otoacoustic emissions (DPOAE) were measured as DP‐grams, enabling a frequency‐specific evaluation of cochlear function.

In order to validate the screening results and, where necessary, perform diagnostic PTA, a reference PTA measurement including air conduction and bone conduction thresholds at 0.25 and 6 kHz was subsequently carried out. In the event of failed screening, audiological diagnostics were conducted immediately. This comprised video otoscopy by the study physician as well as speech audiometry in quiet (with monosyllables and polysyllables) and in noise (with monosyllables) using the Mainz speech audiometry test for children (MATCH test). If PTA was unfeasible or yielded inconclusive results, hearing thresholds were derived either from DPOAE growth functions or auditory brainstem responses (ABR) using broadband chirp stimuli, and/or from auditory steady‐state responses (ASSR) at 0.5, 1, 2 and 4 kHz. Where possible, we classified any hearing loss detected as conductive, sensorineural or mixed conductive/sensorineural hearing loss.

**FIGURE 1:**
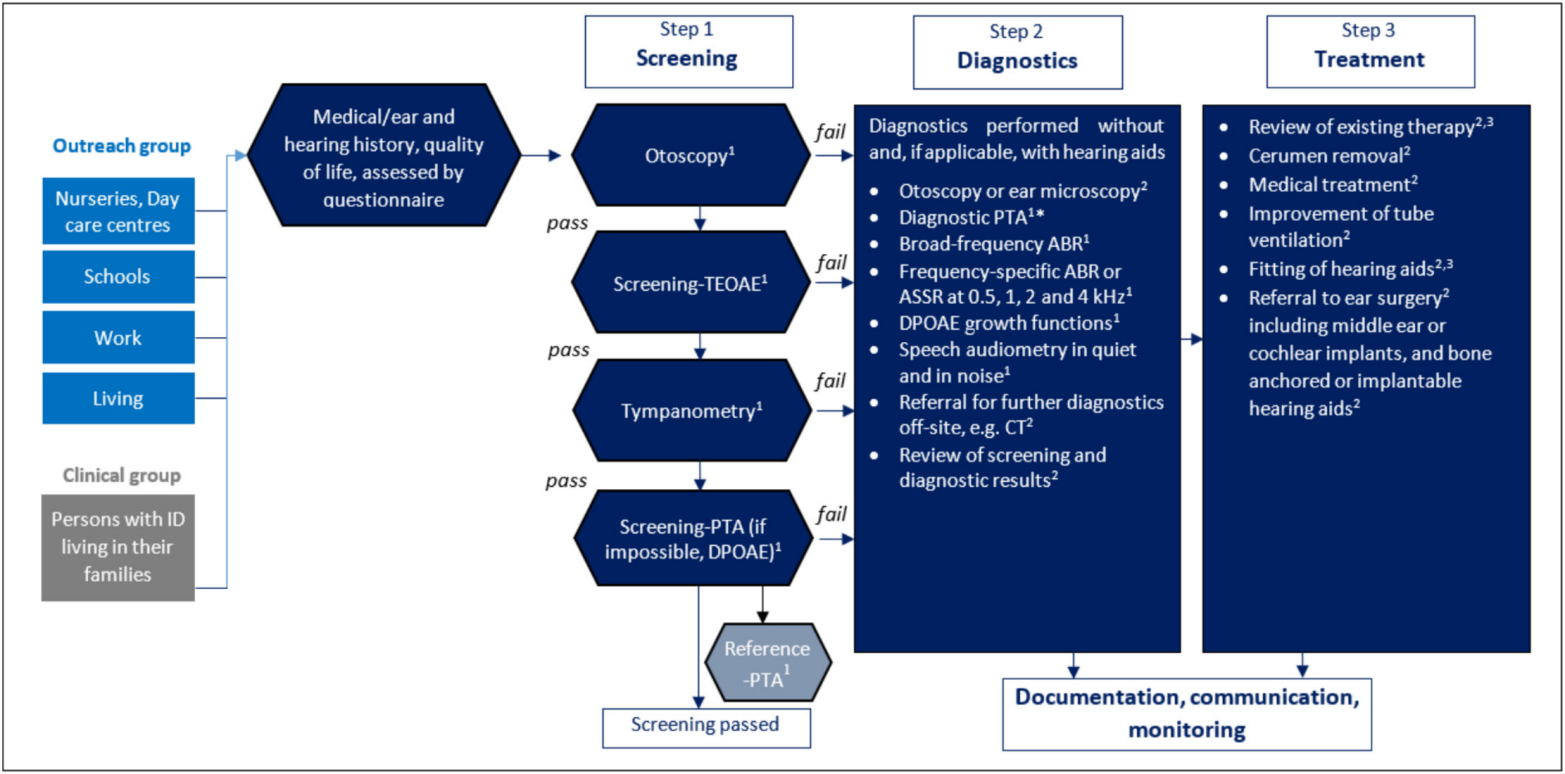
Flow chart of the three‐step *HörGeist* programme. The procedure was performed at both time points T0 and T1.^1^Screening staff; ^2^Physician; ^3^Hearing aid acoustician; *In the event of failed screening, the reference PTA was the diagnostic PTA. ABR, auditory brainstem response; ASSR, auditory steady‐state response; CT, CT scan; DPOAE, distortion‐product otoacoustic emissions; PTA, pure‐tone audiometry; TEOAE, transient‐evoked otoacoustic emissions. Figure adapted from Schwarze et al. (2023).

The supervising study physician provided immediate intervention where necessary, such as cerumen removal, prescriptions of medication, middle ear ventilation balloons or the initiation of hearing aid fitting. The full procedure is outlined in Figure 1.

If on‐site diagnostics were not possible or yielded incomplete or inconclusive findings, participants were sent a medical letter by post containing referrals for further ‘external’ diagnostic evaluation (i.e., at external healthcare facilities). Diagnostic referrals were issued for three main reasons:

(1) follow‐up assessment of identified or pre‐existing ear and/or hearing conditions, such as otitis media with effusion (OME) or impacted cerumen, particularly when on‐site hearing testing suggested an additional hearing loss beyond that caused by the cerumen; (2) further clarification of the type and origin of hearing loss, such as distinguishing between conductive and sensorineural causes; and (3) cases in which inconclusive or unreliable test results—for instance, due to non‐compliance—led to an uncertain assessment of hearing status. A letter was also issued if therapy was recommended but not feasible on‐site. In some cases, a referral for both external diagnostics and therapy was necessary (these were termed ‘diagnostic/therapeutic referrals’), e.g., where further hearing assessment, such as ABR, and cerumen removal were both required. Referrals were also made when it was unclear whether diagnostic evaluation alone or additional treatment would be necessary, such as in cases requiring ongoing monitoring of OME. For external diagnostics, recommended procedures included ear microscopy, recordings of ABR and ASSR, tympanometry, additional audiometric assessments and imaging, specifically thin‐layer CT scans of the petrous bone region. The participants were referred to cooperating clinical facilities specialising in phoniatrics and paediatric audiology, a medical specialty recognised in Germany, in the university hospitals of Cologne, Essen or Münster and a medical practice in Düsseldorf. Further referrals were made to otorhinolaryngological or radiological facilities in the Rhineland region.

**FIGURE 2:**
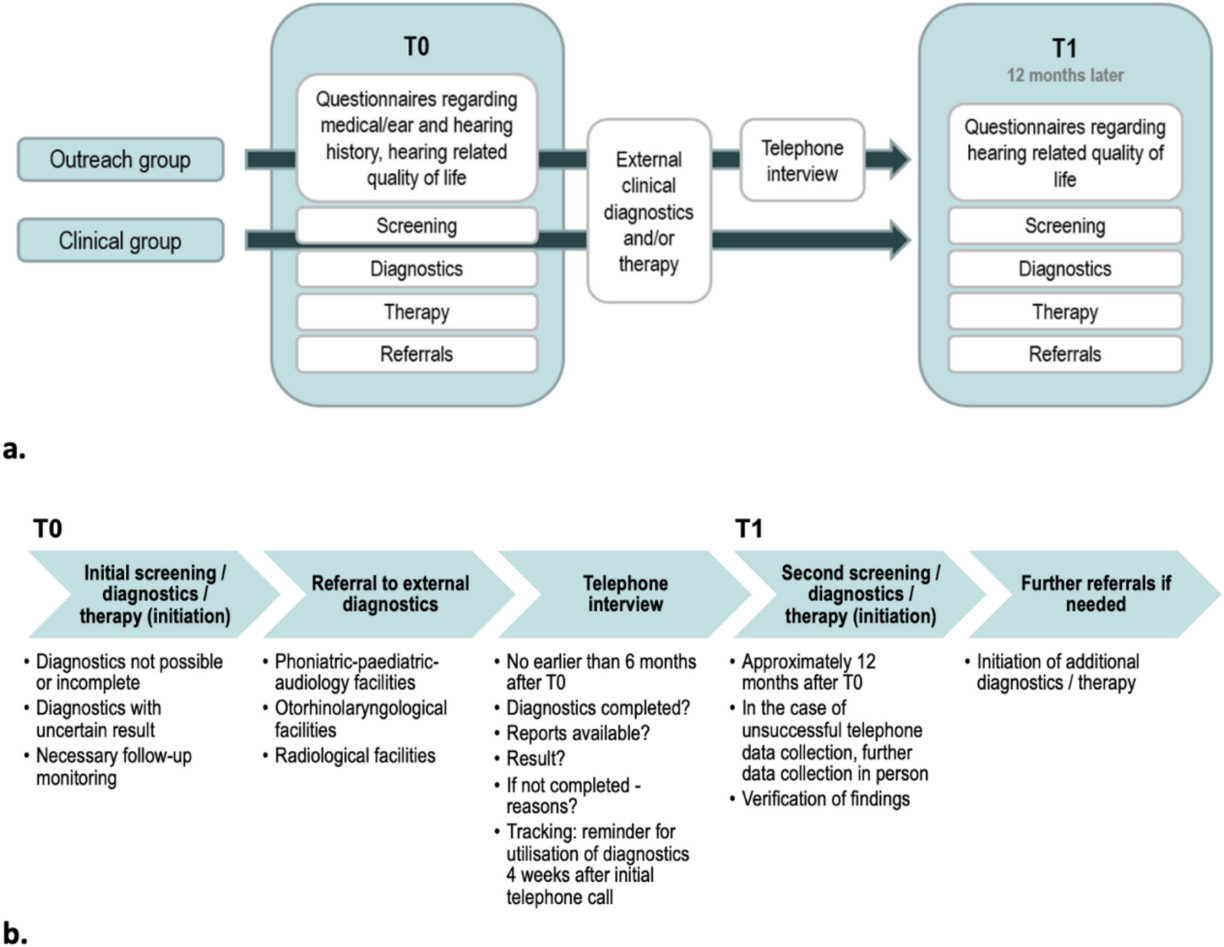
Study protocol and outcome assessment. (a) general procedure; (b) recording of the outcomes of external diagnostics.

For this part of the study assessing the utilisation of external diagnostics in the period between first and second testing, data were collected by telephone interview using a questionnaire developed by the authors (see Supporting Information, HörGeist_Therapy2_.docx). The telephone interviews, conducted no earlier than 6 months after T0, determined how many cases sought or underwent external diagnostics and the results that were obtained. In cases where no external diagnostic appointment was utilised, the reasons for this were recorded according to pre‐established categories for which multiple answers were possible: refusal by caregivers; refusal by participants; no attempt to arrange an appointment; no appointment received; referral letter not received; other reasons/unknown; and additional statements. A subgroup of the sample (47 participants) for whom no appointment had been arranged by the time of the call was encouraged to make use of the referrals and was then contacted again within up to 4 weeks after the initial telephone call to assess whether such tracking could improve referral utilisation. In the event of repeated unsuccessful telephone contact attempts, the data were collected at the follow‐up examination T1. In the case of newly occurring or previously identified but untreated ear and hearing disorders, on‐site diagnostics were conducted again at this second examination and, if necessary, intervention and further referrals were provided. The study procedure is shown in Figure 2a,b.

**FIGURE 3:**
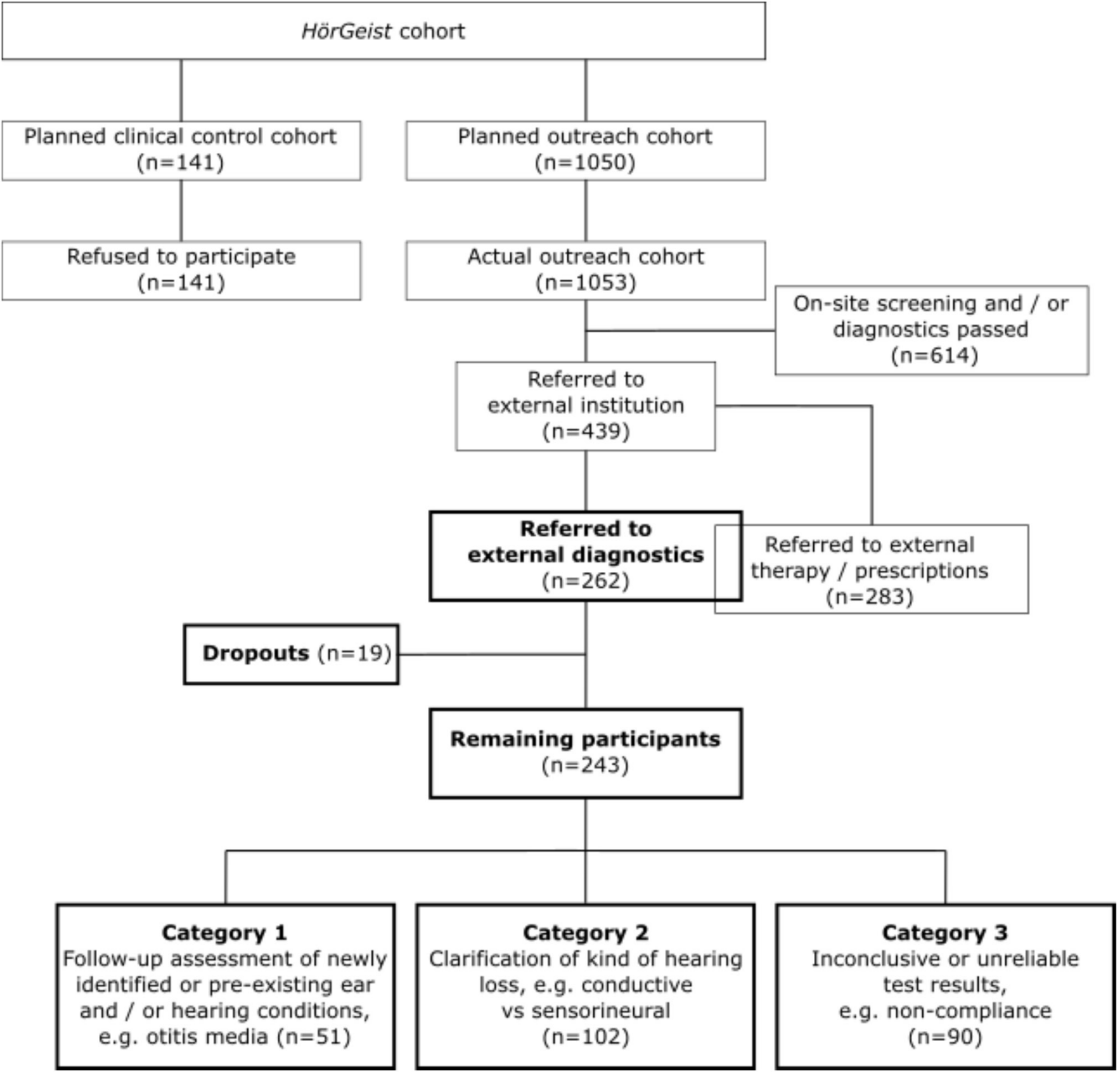
Flow chart illustrating the identification of the external diagnostics substudy cohort within the *HörGeist* study. Boxes shown in bold represent the cohort analysed in the present paper. Some participants referred to external institutions received both diagnostic and therapeutic referrals (illustrated by the overlapping boxes), resulting in totals exceeding the overall number of participants referred to external institutions.

In this part of the study, we focus on participants of the outreach cohort who received referrals for external diagnostics, comprising 262 individuals who therefore form the analysed cohort. This cohort also includes participants who received both diagnostic and therapeutic referrals, as their diagnostic referral component qualified them for inclusion. Participants needing either additional therapy, monitoring of an existing therapy, such as hearing aid fitting, or specific diagnostics prior to cochlear implantation are not reported here. The identification process for the cohort of this part of the study is shown in Figure 3.

## 3 Results

Of the 1053 participants in the outreach group, 262 individuals (24.9%) were referred for further external diagnostics at the T0 assessment. Thereafter, 19 of them (7.3%) dropped out of the study. Reasons for dropouts were death (eight participants), relocation (four participants), medical causes (two participants) and other personal reasons (five participants). Of the remaining 243 participants, 242 were referred to otorhinolaryngological or phoniatric–paediatric–audiology facilities, and six were referred for imaging (double counting possible). Overall, 61 referrals concerned age group 1 (0–5 years), 93 age group 2 (6–17 years) and 94 age group 3 (18–90 years). Across the defined referral categories, 56 referrals were assigned to Category 1, 102 to Category 2 and 90 to Category 3 (total *n* = 248).

In total, only 37.5% of all diagnostic or diagnostic/therapeutic referrals for the 243 participants were realised. Of the 242 participants referred to otorhinolaryngology or phoniatric–paediatric audiology facilities, 91 (37.6%) actually attended. For 22 of them (24.2%), the study team was able to obtain medical records. Two out of six of the individuals referred to radiological facilities (33.3%) received imaging, but no reports were available to the study team. As part of the follow‐up process, 48 of the 159 participants who had not initially utilised the referrals after T0 were contacted again, resulting in 8 (16.7%) of these subsequently scheduling and attending their referral appointments. An age‐related decline in referral uptake was observed, with uptake rates of 50.8%, 41.3% and 24.7% in the three age groups, respectively. For the three predefined referral categories based on the underlying reasons for referral, however, uptake rates did not differ substantially (Category 1: 37.5%, Category 2: 38.2% and Category 3: 36.7%).

In most cases, the diagnostic results from external assessments could not be evaluated by telephone or at T1, as participants and their caregivers were unable to provide information and reported that they had not received any medical reports. The following data are thus drawn from a combination of medical history, findings from both assessment time points and the results obtainable through telephone interviews. At T1, 152 participants (62.6%) of the 243 met the study's criteria for hearing loss. Of these, 117 had already received a diagnosis at T0, while 35 represented new cases. Among the 91 participants without a hearing loss at T1, 36 had previously been diagnosed at T0. Of the 47 tracked individuals, 25 had a hearing loss at T1, including 17 already diagnosed at T0. Of the 22 individuals without a hearing loss at T1, eight had been diagnosed at T0.

A significant number of unattended referrals resulted from a lack of effort to schedule an appointment (32.9%). Furthermore, a large proportion of caregivers declined further external diagnostics (23.2%). The main reasons cited included a perceived lack of necessity, staffing shortages and the prioritisation of other serious diseases affecting the participants. Another significant share of unutilised referrals was due to participants declining further external diagnostics (18.1%). Only 3.9% were unable to secure an appointment due to full bookings.

Analysis across the predefined age groups showed that both caregiver and participant refusal rates increased with higher age categories, whereas no clear age‐related pattern was observed for the remaining reasons for nonutilisation. Across the three predefined referral categories, the proportion of cases reporting no attempt to arrange an appointment varied (5.8%, 13.5% and 13.5%), with refusal rates lowest among caregivers in Category 1 (1.9%) and highest in Category 2 for both caregivers (12.9%) and participants (8.4%). Reasons for non‐attendance, as classified by the categories defined in Section 2, and their frequencies are listed in Table 2 for the overall cohort, the predefined age groups and the predefined referral categories.

**TABLE 2:** Reasons given for not utilising referrals to otorhinolaryngological or phoniatric–paediatric–audiology or radiological facilities shown for the total cohort, by age group and for the previously defined referral categories.

Reasons	Total	Age group 1 (0–5 years)	Age group 2 (6–17 years)	Age group 3 (18–90 years)	Category 1 referral	Category 2 referral	Category 3 referral
No attempt to arrange an appointment	51 (32.9%)	13 (8.4%)	22 (14.2%)	16 (10.3%)	9 (5.8%)	21 (13.5%)	21 (13.5%)
Refusal by caregivers	36 (23.2%)	4 (2.6%)	13 (8.4%)	19 (12.3%)	3 (1.9%)	20 (12.9%)	13 (8.4%)
Referral seen as unnecessary by caregiver	12 (7.7%)	2 (1.3%)	6 (3.9%)	4 (2.6%)	2 (1.3%)	6 (3.9%)	4 (2.6%)
Other serious illness takes priority	7 (4.5%)	2 (1.3%)	0	5 (3.2%)	0	4 (2.6%)	3 (1.9%)
Lack of personnel capacity	3 (1.9%)	0	0	3 (1.9%)	0	3 (1.9%)	0
Unknown	17 (11.0%)	1 (0.6%)	9 (5.8%)	7 (4.5%)	1 (0.6%)	8 (5.2%)	8 (5.2%)
Refusal by participants	28 (18.1%)	2 (1.3%)	5 (3.2%)	21 (13.5%)	5 (3.2%)	13 (8.4%)	10 (6.5%)
No appointment received	6 (3.9%)	3 (1.9%)	2 (1.3%)	1 (0.6%)	1 (0.6%)	2 (1.3%)	3 (1.9%)
Not received doctor's letter with referral	4 (2.6%)	3 (1.9%)	0	1 (0.6%)	1 (0.6%)	2 (1.3%)	1 (0.6%)
Other/unknown	46 (29.7%)	8 (5.2%)	18 (11.6%)	20 (12.9%)	17 (11.0%)	15 (9.7%)	14 (9.0%)
Received only therapy, no diagnostics were carried out	14 (9.0%)	3 (1.9%)	4 (2.6%)	7 (4.5%)	4 (2.6%)	7 (4.5%)	3 (1.9%)
Appointment has not yet taken place, but is planned	4 (2.6%)	2 (1.3%)	2 (1.3%)	0	0	2 (1.3%)	2 (1.3%)
Unknown	28 (18.1%)	3 (1.9%)	12 (7.7%)	13 (8.4%)	13 (8.4%)	6 (3.9%)	9 (5.8%)

*Note:* Percentages refer to the 155 participants of the study cohort who did not utilise diagnostic referrals. As multiple categories of the reasons were possible, percentages do not add to 100; also note that in this table, the number of referrals and not the number of participants are counted.

At T1, in addition to the 19 dropouts from the diagnostic branch, a further 62 dropouts occurred. A total of 972 participants were therefore tested again at T1. Of these 972, only 136 (14.0%) received referrals for further external diagnostics.

## 4 Discussion

The aim of this part of the *HörGeist* study was to determine the extent to which referrals for external diagnostics were necessary and utilised within the framework of an outreach programme for hearing screening, diagnostics, interventions and monitoring and to identify the reasons for nonutilisation of these referrals. Particularly in the light of the known challenges in healthcare provision for individuals with intellectual disabilities, understanding the underlying causes of unmet referrals is essential to improving future diagnostic and therapeutic opportunities.

Only 37.5% of the referrals for external diagnostics were actually followed up on by the participants and their caregivers. The failure to schedule follow‐up appointments was attributable to several factors. In most cases, caregivers—including parents or legal guardians—did not actively pursue an appointment. This may indicate difficulties in understanding the symptoms and consequences of hearing loss, or limited access for caregivers—including parents or legal guardians—to user‐friendly information on diagnostics and treatment options available for the individuals in their care. In many cases, subjective impressions from everyday life were therefore given greater weight than audiometric findings. These circumstances, along with communication barriers that were seemingly normalised by caregivers and attributed mainly to intellectual disability rather than treatable hearing loss, may have further lessened the perceived urgency of follow‐up diagnostic recommendations. Lastly, it can be assumed that the effects of hearing loss may be less noticeable in the often‐simplified verbal communication with individuals with intellectual disabilities than in more complex communicative contexts.

Another relevant factor contributing to the refusal of further diagnostics by caregivers was the presence of other serious diseases among participants, which were prioritised in care and may have consumed resources otherwise needed for hearing diagnostics. This is understandable, considering that examinations were even conducted in hospices and with patients on ventilation. This highlights the competing demands faced by families and care staff when coordinating complex medical care, while also suggesting a potential underestimation of the burden imposed by hearing loss.

An additional barrier reported by caregivers was a lack of available care capacity, both in terms of time and staffing. In many cases, organisational constraints were cited as preventing the completion of diagnostics.

Direct refusal of the examination by the participants themselves was also observed. A particularly challenging factor was compliance during the examination itself. Fear and reluctance regarding medical procedures as well as a lack of understanding of their necessity may have contributed to this. Previous negative experiences with medical care may also have played a role in the unwillingness to attend further diagnostics. If individuals with intellectual disabilities or their caregivers had encountered barriers in the past—such as communication difficulties, scheduling or transportation issues or distressing experiences—their willingness to engage with medical services may be reduced.

An age‐related decline in referral uptake was observed, with 50.8%, 41.3% and 24.7% of referrals completed in the youngest, school‐aged and adult groups. Correspondingly, refusal rates gradually increased among both caregivers and participants. This trend may reflect an age‐related shift in the perceived relevance of hearing diagnostics—considered more essential for younger participants, whose parents often act as advocates for their children's health and language development, while in older individuals, hearing difficulties may be regarded as less critical or more habitual. It may also relate to a decreasing level of dependency on parents and other caregivers with increasing age. Among adults, both a lower acceptance of medical procedures and established communication patterns that may normalise hearing loss could further contribute to this trend. For the remaining categories of non‐attendance, no clear age‐related trend was evident.

Individuals with known or newly diagnosed localised hearing disorders were more inclined to follow up with external diagnostic services, whereas those with hearing loss of uncertain origin or unclear hearing status showed higher refusal rates among both caregivers and participants, and often cited not having attempted to arrange an appointment as a reason for nonuptake. This pattern may reflect differences in perceived necessity and clarity of the referral indication. Individuals with localised or clearly diagnosed hearing disorders may have regarded the referral as more relevant, whereas uncertain test results could have reduced the perceived need for further evaluation. In addition, limited understanding of the findings or doubts regarding their accuracy may have contributed to the higher refusal and nonuptake rates observed in both latter categories. It may further be assumed that when a hearing disorder had already been clearly diagnosed, the therapeutic implications and the potential impact on the individual's or the child's daily life appeared more tangible. In such cases, there may have been a perceived overlap between diagnostic and therapeutic referrals, making the referral seem more meaningful and comprehensible to those affected.

The fact that fewer than 4% of participants reported being unable to obtain an appointment from an external service provider upon request suggests that outpatient and clinical care capacity was generally sufficient. However, this result may be favourably influenced by the inclusion of contact details for cooperating clinics and practices in the referral letters. For broader implementation of such an ear and hearing healthcare programme, ensuring low‐threshold access for this underserved population to necessary medical services is essential.

Only a small number of clinical reports were made available to the study team by caregivers following external diagnostics—often because they had not received such documentation themselves. This illustrates the challenges of effective communication, particularly with the outpatient sector. Combined with the often‐limited medical knowledge among caregivers accompanying a participant, a disrupted flow of information can impede further diagnostics and treatment and may lead to unnecessary duplicate examinations—placing avoidable strain on time, staffing and financial resources. Consequently, the absence of prior diagnostic findings may have led to duplicate diagnostic procedures at T1.

The difference in the proportion of participants receiving referrals for further diagnostics at T0 compared to T1 (24.9% vs. 14.0%) is striking. One possible explanation is the growing experience of the professionals involved in the study with the outreach hearing screening, diagnostics and treatment procedures over time. Additionally, conditions necessitating further diagnostics may already have been identified at T0 and subsequently addressed both diagnostically and therapeutically. These findings highlight the need for continuous training of examiners within a universally implemented, quality‐assured programme in order to improve diagnostic and therapeutic outcomes over time.

Our data also demonstrate that systematic tracking of individuals in need of external diagnostics leads to a higher uptake/follow‐up rate. The benefits of such tracking are well established in newborn hearing screening programmes, where follow‐up tracking of screen‐positive infants significantly reduces the lost‐to‐follow‐up rate in comparison with unguided referral processes (Neumann et al. 2022). This further highlights the importance of close coordination between a screening centre (in our case, the study centre) and a regional network of coproviders in both clinical and outpatient settings. The observed increase in referral uptake among the subsample contacted through systematic telephone tracking (17%) suggests that certain barriers to follow‐up are modifiable through proactive engagement. This underscores the value of low‐cost, structured follow‐up mechanisms to support caregivers and participants in navigating the healthcare system. Future implementations of outreach programmes should therefore incorporate standardised tracking procedures as a central component rather than an optional addition.

Our findings underscore the urgent need for awareness‐raising and educational efforts regarding ear and hearing disorders, their consequences and the necessity of diagnostic, therapeutic and rehabilitative measures. These efforts should target individuals with intellectual disabilities themselves, as well as their caregivers. Given the observed age‐related increase in refusal rates among both caregivers and participants, particular attention should be paid to the support and guidance of older individuals with intellectual disabilities. One possible approach could be the adaptation of the well‐established Australian Active Communication Education (ACE) programme (Hickson et al. 2007). Targeted initiatives, such as public awareness campaigns, the provision of accessible information material (including in Plain Language) or personal counselling, could enhance the willingness to participate in diagnostic and therapeutic measures. Furthermore, tailored support strategies should be implemented in order to address individual barriers to accessing external diagnostics. This could include appointment coordination by the testing staff directly after necessary but incomplete or unperformed on‐site diagnostics.

### 4.1 Limitations

Tracking of participants who had not (yet) undergone external diagnostics was carried out in only around one‐third of relevant cases. For an effective universal programme, standardised tracking of all individuals who failed or could not complete screening—and did not receive full on‐site diagnostics—would be essential, in a manner similar to newborn hearing screening.

External findings could not be reliably obtained in many cases, owing to limited access to results and insufficient medical understanding among participants and their caregivers. A universally implemented and regularly conducted programme must therefore ensure standardised information delivery to caregivers, including easily understandable recommendations for further diagnostic and therapeutic steps—ideally mediated by a hearing screening centre.

## 5 Conclusion

In our study, slightly more than one third of individuals with intellectual disabilities who required further clinical diagnostic evaluation—following either failed or incomplete outreach hearing screening or an incomplete on‐site assessment—actually received the recommended examinations. By contrast, there was a complete absence of participation in the control group, which further emphasises the significance of this finding. On the one hand, and particularly in light of this stark difference, the results support our recommendation to conduct ear and hearing assessments for people with intellectual disabilities in their familiar living environments rather than in clinical settings. On the other hand, the findings highlight the pressing need to raise awareness of the widespread underdiagnosis and undertreatment of ear and hearing disorders in this population—including their symptoms and consequences—among caregivers, affected individuals, medical and audiological professionals and the general public.

Regular training of medical and audiological staff involved in such programmes is essential in order to ensure optimal test performance and therapeutic outcomes. Close networking between outpatient and clinical service providers, facilitated by a hearing screening centre, is vital in order to improve information flow. The implementation of such an ear and hearing healthcare programme should include standardised tracking of diagnostic progress in order to reduce the lost‐to‐follow‐up rate. Targeted support measures should also be developed in order to lower existing access barriers to external diagnostics—for instance, through direct appointment scheduling initiated within the programme.

## Funding

This study was funded by the Innovation Fund of the Federal Joint Committee (G‐BA, Germany), grant number 01NVF18038.

## Ethics Statement

This was reviewed and approved by the Ethics Committee of the Medical Association of Westphalia‐Lippe and the University of Münster (No. 2020‐843‐f‐S).

## Conflicts of Interest

The authors declare no conflicts of interest.

## Supporting information


**Data S1:** Supporting information.

## Data Availability

The data used for the statistical analyses are available from the corresponding author upon reasonable request.

## References

[jir70078-bib-0001] Anders, P. L. , and E. L. Davis . 2010. “Oral Health of Patients With Intellectual Disabilities: A Systematic Review.” Special Care in Dentistry 30, no. 3: 110–117. 10.1111/j.1754-4505.2010.00136.x. 20500706

[jir70078-bib-0002] Bonino, A. Y. , D. Mood , and M. S. Dietrich . 2024. “Rethinking the Accessibility of Hearing Assessments for Children With Developmental Disabilities.” Journal of Autism and Developmental Disorders 55, no. 10: 3711–3721. 39023803 10.1007/s10803-024-06461-9 PMC12476404

[jir70078-bib-0003] Bower, C. , H. Leonard , and B. Petterson . 2000. “Intellectual Disability in Western Australia.” Journal of Paediatrics and Child Health 36, no. 3: 213–215. 10.1046/j.1440-1754.2000.00480.x. 10849218

[jir70078-bib-0004] Danyluk, A. , and R. Jacob . 2023. “Hearing Loss Diagnosis and Management in Adults With Intellectual and Developmental Disabilities.” Advances in Medicine 2023, no. 1: 6825476. 37251596 10.1155/2023/6825476 PMC10225271

[jir70078-bib-0005] Esbensen, A. J. 2010. “Health Conditions Associated With Aging and End of Life of Adults With Down Syndrome.” In International Review of Research in Mental Retardation, vol. 39, 107–126. Elsevier. 21197120 10.1016/S0074-7750(10)39004-5 PMC3010180

[jir70078-bib-0006] Evenhuis, H. M. 1996. “Dutch Consensus on Diagnosis and Treatment of Hearing Impairment in Children and Adults With Intellectual Disability.” Journal of Intellectual Disability Research 40: 451–456. 8906532 10.1046/j.1365-2788.1996.788788.x

[jir70078-bib-0007] Evenhuis, H. M. , M. Theunissen , I. Denkers , H. Verschuure , and H. Kemme . 2001. “Prevalence of Visual and Hearing Impairment in a Dutch Institutionalized Population With Intellectual Disability.” Journal of Intellectual Disability Research 45, no. 5: 457–464. 10.1046/j.1365-2788.2001.00350.x. 11679051

[jir70078-bib-0008] García, J. C. , E. Díez , D. Z. Wojcik , and M. Santamaría . 2020. “Communication Support Needs in Adults With Intellectual Disabilities and Its Relation to Quality of Life.” International Journal of Environmental Research and Public Health 17, no. 20: 7370. 10.3390/ijerph17207370. 33050216 PMC7601275

[jir70078-bib-0009] Haile, L. M. , K. Kamenov , P. S. Briant , et al. 2021. “Hearing Loss Prevalence and Years Lived With Disability, 1990‐2019: Findings From the Global Burden of Disease Study 2019.” Lancet 397, no. 10278: 996–1009. 10.1016/S0140-6736(21)00516-X. 33714390 PMC7960691

[jir70078-bib-0010] Herer, G. R. 2012. “Intellectual Disabilities and Hearing Loss.” In Communication Disorders Quarterly, vol. 33(4), 252–260. SAGE Publications Sage CA.

[jir70078-bib-0011] Heß, C. , F. Rosanowski , U. Eysholdt , and M. Schuster . 2005. “Hörvermögen bei Kindern und Jugendlichen mit Down‐Syndrom.” HNO 53: 227–232. 10.1007/s00106-004-1205-y. 15657750

[jir70078-bib-0012] Hey, C. , S. Fessler , N. Hafner , B. P. Lange , H. A. Euler , and K. Neumann . 2014. “High Prevalence of Hearing Loss at the Special Olympics: Is This Representative of People With Intellectual Disability?” Journal of Applied Research in Intellectual Disabilities 27, no. 2: 125–133. 23610001 10.1111/jar.12057

[jir70078-bib-0013] Hickson, L. , L. Worrall , and N. Scarinci . 2007. “A Randomized Controlled Trial Evaluating the Active Communication Education Program for Older People With Hearing Impairment.” Ear and Hearing 28, no. 2: 212–230. 10.1097/AUD.0b013e31803126c8. 17496672

[jir70078-bib-0014] Hild, U. , C. Hey , U. Baumann , J. Montgomery , H. A. Euler , and K. Neumann . 2008. “High Prevalence of Hearing Disorders at the Special Olympics Indicate Need to Screen Persons With Intellectual Disability.” Journal of Intellectual Disability Research 52, no. june: 520–528. 10.1111/j.1365-2788.2008.01059.x. 18410317

[jir70078-bib-0015] Kreicher, K. L. , F. W. Weir , S. A. Nguyen , and T. A. Meyer . 2017. “Characteristics and Progression of Hearing Loss in Children With Down Syndrome.” Journal of Pediatrics 193: 27–33.e2. 10.1016/j.jpeds.2017.09.053. 29174076

[jir70078-bib-0016] Kumar Sinha, A. , J. K. Montgomery , G. R. Herer , and D. L. McPherson . 2008. “Hearing Screening Outcomes for Persons With Intellectual Disability: A Preliminary Report of Findings From the 2005 Special Olympics World Winter Games.” International Journal of Audiology 47, no. 7: 399–403. 18574777 10.1080/14992020801889535

[jir70078-bib-0017] Lennox, N. , M. Taylor , T. Rey‐Conde , C. Bain , D. M. Purdie , and F. Boyle . 2005. “Beating the Barriers: Recruitment of People With Intellectual Disability to Participate in Research.” Journal of Intellectual Disability Research 49, no. 4: 296–305. 10.1111/j.1365-2788.2005.00618.x. 15816817

[jir70078-bib-0018] Lennox, N. , R. Ware , C. Bain , G. M. Taylor , and S. A. Cooper . 2011. “Effects of Health Screening for Adults With Intellectual Disability: A Pooled Analysis.” British Journal of General Practice 61, no. 584: 193–196. 10.3399/bjgp11X561186 PMC3047312 21375903

[jir70078-bib-0019] Määttä, T. , J. Määttä , T. Tervo‐Määttä , A. Taanila , M. Kaski , and M. Iivanainen . 2011. “Healthcare and Guidelines: A Population‐Based Survey of Recorded Medical Problems and Health Surveillance for People With Down Syndrome.” 36, no. 2: 118–126. 10.1080/13668250.2011.570253 21501111

[jir70078-bib-0020] Mackenzie, I. , and A. Smith . 2009. “Deafness—The Neglected and Hidden Disability.” Annals of Tropical Medicine and Parasitology 103, no. 7: 565–571. 19825278 10.1179/000349809X12459740922372

[jir70078-bib-0021] Malt, E. A. , R. C. Dahl , T. M. Haugsand , et al. 2013. Health and Disease in Adults With Down Syndrome. Tidsskrift for Den norske legeforening. 10.4045/tidsskr.12.0390 23381164

[jir70078-bib-0033] Meuwese‐Jongejeugd, A. , M. Vink , B. van Zanten , et al., 2006. “Prevalence of Hearing Loss in 1598 Adults With an Intellectual Disability: Cross‐Sectional Population Based Study.” International Journal of Audiology 45, no. 11: 660–669. 10.1080/14992020600920812. 17118908

[jir70078-bib-0022] Michels, T. C. , and P. Angeles . 2019. “Hearing Loss in Adults: Differential Diagnosis and Treatment.” 100(2). 31305044

[jir70078-bib-0023] Neumann, K. , P. Böttcher , and A. Euler . 2006. “Effectiveness and Efficiency of a Universal Newborn Hearing Screening in Germany.” 440–455. 10.1159/000095004. 17108701

[jir70078-bib-0024] Neumann, K. , G. Dettmer , H. A. Euler , et al. 2006. “Auditory Status of Persons With Intellectual Disability at the German Special Olympic Games.” International Journal of Audiology 45, no. 2: 83–90. 10.1080/14992020500376891. 16566246

[jir70078-bib-0025] Neumann, K. , P. Mathmann , S. Chadha , H. A. Euler , and K. R. White . 2022. “Newborn Hearing Screening Benefits Children, but Global Disparities Persist.” Journal of Clinical Medicine 11, no. 1: 271. 10.3390/jcm11010271. 35012010 PMC8746089

[jir70078-bib-0026] Nightengale, E. , P. Yoon , K. Wolter‐Warmerdam , D. Daniels , and F. Hickey . 2017. “Understanding Hearing and Hearing Loss in Children With Down Syndrome.” American Journal of Audiology 26, no. 3: 301–308. 10.1044/2017_AJA-17-0010. 28854301

[jir70078-bib-0027] Riha, R. L. , A. Singh , E. A. Hill , H. Evans , and D. O'Regan . 2024. “Sleep‐Disordered Breathing in Children and Adults With Intellectual Disability: Mind the Gap!” Thorax 79, no. 11: 1099–1107. 10.1136/thorax-2023-220032. 38937106

[jir70078-bib-0028] Savvas, E. , E. Dimitrakopoulou , H. A. Euler , et al. 2025. “Hearing Threshold Estimation With Distortion Product Otoacoustic Emission Growth Functions in People With Intellectual Disabilities in an Outreach Setting.” Journal of Intellectual Disability Research 69, no. 12: 1474–1485. 10.1111/jir.70043. 40976998 PMC12580478

[jir70078-bib-0029] Schwarze, K. , P. Mathmann , K. Schäfer , et al. 2023. “Effectiveness and Costs of a Low‐Threshold Hearing Screening Programme (HörGeist) for Individuals With Intellectual Disabilities: Protocol for a Screening Study.” BMJ Open 13, no. 5: 1–8. 10.1136/bmjopen-2022-070259. PMC10201260 37202136

[jir70078-bib-0030] Shady, K. , S. Phillips , and S. Newman . 2022. “Barriers and Facilitators to Healthcare Access in Adults With Intellectual and Developmental Disorders and Communication Difficulties: An Integrative Review.” Review Journal of Autism and Developmental Disorders 11, no. 1: 39–51. 10.1007/s40489-022-00324-8. PMC9148936 35669718

[jir70078-bib-0031] Sullivan, W. F. , and J. Heng . 2018. “Supporting Adults With Intellectual and Developmental Disabilities to Participate in Health Care Decision Making.” Canadian Family Physician 64, no. Suppl 2: 32–36. 29650742 PMC5906782

[jir70078-bib-0032] Trudeau, S. , S. Anne , T. Otteson , B. Hopkins , R. Georgopoulos , and C. Wentland . 2021. “Diagnosis and Patterns of Hearing Loss in Children With Severe Developmental Delay.” American Journal of Otolaryngology 42, no. 3: 102923. 33486206 10.1016/j.amjoto.2021.102923

[jir70078-bib-0034] Willems, M. , F. Acke , B. Lannon , L. Leyssens , L. Maes , and L. Marks . 2022. “Global Data on Ear and Hearing Screening in an Intellectual Disability Population.” American Journal on Intellectual and Developmental Disabilities 127, no. 2: 125–134. 10.1352/1944-7558-127.2.125. 35180777

[jir70078-bib-0035] Yoshinaga‐Itano, C. , V. Manchaiah , and C. Hunnicutt . 2021. “Outcomes of Universal Newborn Screening Programs: Systematic Review.” Journal of Clinical Medicine 10: 2784. 10.3390/jcm10132784. 34202909 PMC8268039

[jir70078-bib-0036] Yoshinaga‐Itano, C. , A. L. Sedey , D. K. Coulter , and A. L. Mehl . 1998. “Language of Early‐ and Later‐Identified Children With Hearing Loss.” Pediatrics 102, no. 5: 1161–1171. 10.1542/peds.102.5.1161. 9794949

[jir70078-bib-0037] Zielonkowski, S. , P. Mathmann , A. Naghipour , et al. 2025. “Evaluating the Hearing‐Related Quality of Life in People With Intellectual Disabilities.” Journal of Intellectual Disability Research 69: 1413–1424. 10.1111/jir.70036. 40963427 PMC12580484

