# Supplementary material for: Clinical Diagnostics After Failed Hearing Screening in People With Intellectual Disabilities Do Not Often Take Place
Source: J Intellect Disabil Res. 2026 Jan 16;70(4):384–94. doi: 10.1111/jir.70078 (PMC12950632; doi:10.1111/jir.70078)
Supplement: Supplementary file 1 — Data S1: Supporting information. [file JIR-70-384-s001.docx]

**Recommended diagnostics/therapy based on HörGeist screening 1 yes ☐ no ☐**

**Hearing aid provision on** (date)

☐ Optimisation of existing provision   ☐ right ☐ left ☐ both sides

☐ Initial provision for a newly detected hearing loss   ☐ right ☐ left ☐ both sides

☐ New prescription due to outdated hearing aid    ☐ right ☐ left ☐ both sides

☐ New prescription due to insufficient provision   ☐ right ☐ left ☐ both sides

Provision completed  ☐ yes ☐ no

By whom/where? __________________________________

Result: ________________________________________

If not completed, reason:

☐ Referral letter not received

☐ No appointment received

☐ No attempt to arrange an appointment

☐ Refused by participant

☐ Refused by caregiver

☐ Other: _________________________________________

**Initiation of cochlear implant treatment took place on** (date)

☐ right ☐ left ☐ both sides

Provision completed  ☐ yes ☐ no

By whom/where? __________________________________

Result: ________________________________________

If not completed, reason:

☐ Referral letter not received

☐ No appointment received

☐ No attempt to arrange an appointment

☐ Refusal by participant

☐ Refusal by caregiver

☐ Other: _________________________________________

**Prescription issued on**: ________

☐ Otovent ☐ Ear drops ☐ Antibiotic ☐ FM system

☐ Other: _____________________________________________________

Result: ________________________________________

If not completed, reason:

☐ Referral letter not received

☐ Refusal by participant

☐ Refusal by caregiver

☐ Other: _________________________________________

**Further otorhinolaryngological assessment/treatment took place on (**date)

Diagnostics completed  ☐ yes ☐ no

Report available  ☐ yes ☐ no

By whom/where? __________________________________

Result: ________________________________________

If not completed, reason:

☐ Referral letter not received

☐ No appointment received

☐ No attempt to arrange an appointment

☐ Refusal by participant

☐ Refusal by caregiver

Other: _________________________________________

**Further phoniatric-paediatric-audiological** **assessment/treatment took place on** (date)

Diagnostics completed  ☐ yes ☐ no

Report available  ☐ yes ☐ no

By whom/where? __________________________________

Result: ________________________________________

If not completed, reason:

☐ Referral letter not received

☐ No appointment received

☐ No attempt to arrange an appointment

☐ Refusal by participant

☐ Refusal by caregiver

Other: _________________________________________

**Imaging carried out on** (date)

☐ CT scan ☐ MRI ☐ Other: ______________________

Diagnostics completed  ☐ yes ☐ no

Report available  ☐ yes ☐ no

By whom/where? __________________________________

Result: ________________________________________

If not completed, reason:

☐ Referral letter not received

☐ No appointment received

☐ No attempt to arrange an appointment

☐ Refusal by participant

☐ Refusal by caregiver

Other: _________________________________________

**Other assessment carried out (please specify): ____________**(date)

Diagnostics completed  ☐ yes ☐ no

Report available  ☐ yes ☐ no

By whom/where? __________________________________

Result: ________________________________________

If not completed, reason:

☐ Referral letter not received

☐ No appointment received

☐ No attempt to arrange an appointment

☐ Refusal by participant

☐ Refusal by caregiver

Other: _________________________________________

………………………………………………………………………………………………………………..

**Supervision by study physician**  ☐ yes  Date: ________  ☐ no

**Further therapy and therapy monitoring required**  ☐ yes ☐ no

If yes:

☐ Within the HörGeist programme (participant’s living environment)   ☐ yes ☐ no

______________________________________________________

**All therapy-related information is complete and correct.**

Date: ________

Signature of study physician: __________________________
